# Proteomics in Alcohol Research

**Published:** 2002

**Authors:** Helen Anni, Yedy Israel

**Affiliations:** Helen Anni, Ph.D., is a research assistant professor in the Department of Pathology, Anatomy, and Cell Biology, and in the Alcohol Research Center, Thomas Jefferson University, Philadelphia, Pennsylvania. Yedy Israel, Ph.D., is a professor in the Department of Pharmacology and Toxicology at the Faculty of Chemical and Pharmaceutical Sciences, University of Chile, and at the Millennium Institute of Advanced Studies in Cell Biology and Biotechnology, Santiago, Chile. He is also a professor in the Department of Pathology, Anatomy, and Cell Biology, and in the Alcohol Research Center, Thomas Jefferson University, Philadelphia, Pennsylvania

**Keywords:** proteins, protein metabolism, physiological AODE (alcohol and other drug effects), alcoholic beverage, genetic mapping, gene expression, AODR (alcohol and other drug related) biological markers, signal transduction, field separation method, research agenda

## Abstract

The proteome is the complete set of proteins in an organism. It is considerably larger and more complex than the genome—the collection of genes that encodes these proteins. Proteomics deals with the qualitative and quantitative study of the proteome under physiological and pathological conditions (e.g., after exposure to alcohol, which causes major changes in numerous proteins of different cell types). To map large proteomes such as the human proteome, proteins from discrete tissues, cells, cell components, or biological fluids are first separated by high-resolution two-dimensional electrophoresis and multidimensional liquid chromatography. Then, individual proteins are identified by mass spectrometry. The huge amount of data acquired using these techniques is analyzed and assembled by fast computers and bioinformatics tools. Using these methods, as well as other technological advances, alcohol researchers can gain a better understanding of how alcohol globally influences protein structure and function, protein–protein interactions, and protein networks. This knowledge ultimately will assist in the early diagnosis and prognosis of alcoholism and the discovery of new drug targets and medications for treatment.

The proteome is defined as the collection of all the proteins in an organism. The human proteome has been estimated to have over 1 million proteins, which are found in the approximately 250 different cell types under various physiological and pathological conditions. Compared with the genome—the entire set of genes that encode the proteins—the proteome is much larger and more complex. Several reasons contribute to the greater size and complexity of the proteome:

- The genetic information contained in some genes can be converted into more than one protein per gene through a process called differential splicing.
- A plethora of changes in protein structure, called post-translational modifications (PTMs), can occur after protein synthesis.
- Many proteins do not act alone but interact with other proteins to transmit biological signals and regulate cell function.

Thus, unlike the genome, which consists of a fixed number of genes that are turned on or off, the proteome is a more dynamic system. External stimuli, such as exposure to alcohol, also can affect numerous proteins in terms of their abundance, and the types of PTMs they undergo. In addition, alcohol exposure may shift the types of proteins that are produced in a cell—for example, favoring regulatory proteins that add phosphate groups to other proteins (i.e., kinases) to modulate protein activity over proteins that remove those phosphate groups (i.e., phosphatases).

The term “proteomics” refers to the large-scale analysis of protein structure, function, and interactions. In the pre-proteomics era, researchers could study only one or a few proteins at a time. With proteomic tools, however, large numbers of proteins can be studied at the same time. For example, for organisms with small proteomes (e.g., bacteria or yeast), investigators can analyze almost all proteins present in the organism simultaneously. For larger proteomes, such as the human proteome, scientists must reduce the number of proteins to be investigated concurrently by focusing on the proteins found in certain tissues, cell types, cell components, biochemical pathways, or other groupings. These data can later be reassembled to derive the entire proteome. Through this process, proteomics promises to elucidate the regulation of protein networks in health and disease and to allow the discovery of a new generation of drug targets and medications for molecular medicine.

This article reviews the emerging field of proteomics in alcohol research. After introducing the basic concepts of proteomics and discussing the importance of studying entire proteomes, the article describes the most important tools used in proteomics research and in the analysis of protein–protein interactions. The article concludes with a summary of potential applications of proteomics to alcohol research.

## From Genomics to Proteomics

The Human Genome Project recently presented a draft map of virtually all the 35,000–45,000 genes found in humans. Already, some of these genes have been associated with the susceptibility to and inheritance of certain diseases. More associations will certainly be discovered in the future as the research focus gradually shifts from structural maps of genes (i.e., the arrangement of genes on the chromosomes) to the area of functional genomics—the study of the initial gene products, the messenger RNA (mRNA) molecules. (For more information on the conversion of genetic information into gene products, see the textbox “Gene Expression.”)

**Gene Expression**

When a gene is switched on (i.e., expressed), the DNA segment containing that gene is copied into a molecule called ribonucleic acid (RNA). This process, which occurs primarily in the cell nucleus, is called transcription. In higher organisms, proteins called transcription factors regulate gene expression. These factors are modular—they consist of a binding domain that interacts with a DNA region near the gene (i.e., the promoter) and an activating domain which interacts with the enzyme that generates the RNA. Several types of RNA exist in the cell. One type, the messenger RNA (mRNA), serves as an intermediary molecule that relays the genetic information from the nucleus to the cytoplasm. mRNA is obtained from the original RNA transcript through a process called splicing. During this process, those RNA sections that do not contain information for the final protein (i.e., the introns) are cut out of the original transcript. The remaining sections of the original transcript (i.e., the exons), which contain the information for the final protein, are then assembled to generate the mRNA.

Depending on the tissue or disease state studied, differential splicing may occur. This means that enzymes in the cell can process an original RNA molecule into different mRNA molecules by combining alternative exons. The resulting mRNAs encode different proteins.

The spliced mRNA moves to the cytoplasm, where it serves as a template for protein production. Two types of RNA—transfer RNA (tRNA) and ribosomal RNA (rRNA)—are components of the cell’s protein production machinery. During this process, which is called translation, protein building blocks—the amino acids—are assembled into long chains according to the specification encoded in the mRNA. The amino acid chain then folds itself into a specific three-dimensional shape. Individual amino acids in the protein then may undergo post-translational modification (PTM) by stable, irreversible addition of various chemical groups.

There are several reasons why the study of the proteins produced by a cell can be more useful than traditional genetic analyses for understanding the processes contributing to the cell’s normal and pathologic functioning. These reasons include the following:

- Not every gene in the genome is actively producing mRNA transcripts at any given moment, and even the presence of mRNA molecules does not ensure that functional proteins will be synthesized (Pradet-Balade et al. 2001).
- Differential RNA splicing occurs with many genes. In certain cells or under certain conditions, an initial RNA transcript of a DNA region can be “cut and pasted” (i.e., spliced) in various ways to create different mRNA molecules encoding different proteins.
- The number of mRNA copies does not always reflect the number of protein molecules that will be made—that is, one mRNA molecule may be used to produce one copy of the corresponding protein or several copies. For example, Celis and colleagues (2000) studied the abundance of the mRNA and the corresponding proteins for 19 gene products in the human liver. Their analysis found a correlation between mRNA and protein levels of 48 percent, a value that is in the middle of the range between perfect correlation (100 percent) and no correlation (0 percent).
- Even after the proteins have been synthesized they may not assume their correct three-dimensional structure, or they may be transported to the wrong area of the cell, so that no functional protein is available to the cell in the area where it is needed.

These findings indicate that only by examining proteins directly can one measure their relative abundance as well as their function, localization within the cell, and interactions with other proteins in complexes. Thus, studies of proteins are crucial for elucidating the cellular role of gene products.

### PTMs as a Source of Protein Diversity

In every human cell, only a fraction of the genes are switched on at any given time, producing no more than 6,000 primary proteins in a process called translation (see the textbox “Gene Expression”). However, several hundred types of PTMs occur, greatly augmenting the number of proteins actually found in cells (Gooley and Packer 1997). These modifications, which involve the stable, irreversible addition of non–amino acid chemical groups to primary translation products, occur in a large proportion of proteins. (Common types of PTMs are listed in the textbox “Types of Post-Translational Modifications.”) In some instances one can already predict what PTMs a protein may undergo by looking at the DNA sequence of a gene and deducing characteristic amino acid sequence motifs. In many cases, however, such predictions are not possible, and one has to study the actual protein to determine what type of PTM has occurred, if any. These modifications can result in an enormous degree of protein diversity. For example, glycosylation—the addition of sugar chains of varying lengths and compositions—of 1 unmodified protein at 3 sites can generate 11,520 protein variants.

**Types of Post-Translational Modifications (PTMs)**

The most common PTMs are:

- Glycosylation—the addition of one or more sugar molecules, which may involve more than one type of sugar
- Phosphorylation—the addition of phosphate groups
- Myristoylation and prenylation—the addition of certain fatty acids
- Ubiquitination—the addition of one or more ubiquitin molecules, which marks the protein for degradation
- Addition of a prosthetic group (e.g., heme in hemeproteins, such as hemoglobin), which is required for the protein’s function)
- Addition of a certain chemical bond (i.e., a disulfide bond) between two sulfur-containing amino acids
- Addition of a target leader sequence (a small removable peptide) at the beginning of the protein chain to allow the protein to be imported to or exported from cell organelles (e.g., nuclei and mitochondria)
- Assembly of individual subunits into a larger structure (e.g., the combination of four protein chains to form hemoglobin), which enhances overall activity.

PTMs are involved in a variety of developmental and pathophysiological conditions. They are also of great interest in alcohol research. For example, some products of alcohol metabolism (e.g., alpha-hydroxyethyl radicals, acetaldehyde, and lipid peroxides) generate PTMs, and alcohol consumption influences the extent of certain PTMs (see “What Is Ahead for Alcohol Research,” below, for more information on these processes).

Because proteins perform most functions in a cell, proteomics analyses are of paramount importance. The objective of proteomics is not just to list all proteins in a cell, tissue, organ, or organism. Instead, this research aims to determine the proteins’ functions and interacting partners under various physiological and pathological conditions as well as to identify new therapeutic targets, improved medications, and clinical markers that may be useful for diagnosis. For excellent reviews of proteomics, see Pandey and Mann (2000) and Liebler (2001).

## Basic Tools of Proteomics

**Figure 1:**
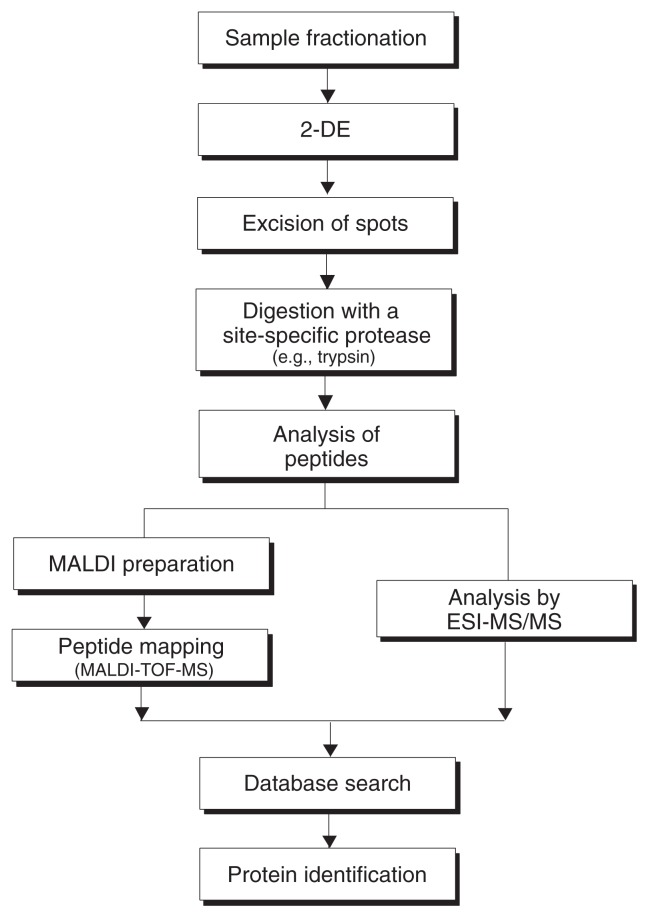
Flowchart showing the process of protein identification through a combination of two-dimensional gel electrophoresis (2–DE) with mass spectroscopy (MS). If the matrix-assisted laser desorption/ionization time-of-flight mass spectroscopy (MALDI–TOF MS) approach does not result in protein identification, additional analyses, such as electrospray ionization (ESI) combined with at least two steps of MS, may be used.

The field of proteomics has been expanding in recent years with the discovery of a multitude of new proteins and the development of appropriate tools for large-scale analysis. This section describes some routine techniques as well as more recent promising tools used in the production, separation, structural and functional characterization, and quantification of proteins. Figure 1 summarizes the steps involved in a classical approach for characterizing proteins in a biological sample, which are described in more detail in the following sections.

To identify and characterize the daunting number of proteins found in an organism, researchers use a “divide to conquer” strategy, focusing on the proteins contained in a given tissue, cell type, cell structure, or biological fluid. A tissue sample (e.g., biopsy material from liver tissue) first is ground up and mixed with various chemicals to obtain a cellular extract from which other biomolecules are removed. The proteins in this extract are then fractionated into less complex mixtures, and the proteins in the mixtures are subsequently separated. For the initial fractionation, researchers typically use multidimensional liquid chromatography (LC). To separate proteins in mixtures, a commonly used technique is two-dimensional gel electrophoresis (2–DE). For increased separation power, both techniques can be combined. The separated proteins then are channeled to mass spectrometers, where their mass and amino acid sequence are determined so that the proteins can be identified. Modern mass spectrometry (MS) in its different variations is the workhorse of proteomics and can provide accurate information about the structure of almost every protein.

### Separation and Identification Techniques

#### LC

Liquid chromatography methods for separating proteins rely on the differences between molecules in how they behave in a liquid phase (i.e., a solution) that moves through a stationary phase (i.e., a solid support). The degree to which the molecules are held back by the solid phase (i.e., the partition between the solid and liquid phases) can depend on the size, electrical charge, or other property of the proteins. In high performance liquid chromatography (HPLC), the solid phase is contained in a narrow column, and the solution passes the sample through it under high pressure. Proteins that interact with the solid phase will spend a greater amount of time in the column than will proteins that stay predominantly in the liquid phase and therefore pass through the column faster. As the components exit the column, they can be collected for further analysis. For example, consecutive fractions (i.e., buffer drops that contain the separated proteins) coming out of the column can be collected and passed directly to an attached mass spectrometer.

#### 2–DE

For this technique, a protein mixture is applied at one end of a flat sheet of a gelatinelike material, the polyacrylamide gel. This gel can be considered a “map” with east, west, north, and south sides. The gel is submerged in a specific solution, and an electrical current is applied to two opposite ends of the gel (e.g., east and west). Under the influence of this current, the proteins start to migrate through the gel (e.g., east to west), with different proteins migrating at different speeds, depending on their total electrical charges (i.e., their isoelectric point). Once this separation is complete, the gel is turned by 90 degrees, submerged in a different solution, and again exposed to an electric current. This time, however, the proteins migrate north to south and their speed is determined by their size. At the end of this separation run, each protein has a specific location on the map.

**Figure 2:**
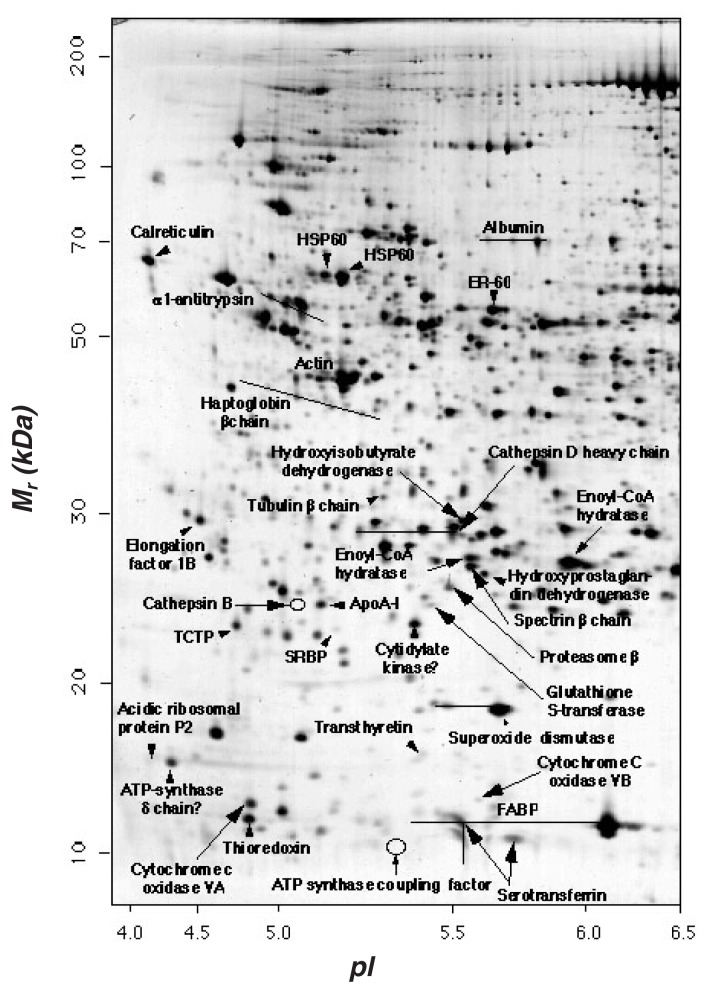
Example of a separation of human liver proteins by two-dimensional gel electrophoresis. Proteins were separated according to their isoelectric point (*pI* 4.0–6.5, acidic proteins) on the X axis, and their molecular weight (*M_r_* 10–200 *kDa*) on the Y axis. Known proteins are labeled, but each spot might represent more than one unresolved protein. Multiple spots adjacent to each other have the same label because they represent post-translational modifications of the same protein, having the same molecular weights but different isoelectric points. SOURCE: Taken from SWISS-2DPAGE maps at http://us.expasy.org/ch2dothergifs/publi/liver-acidic.gif

Under optimal conditions, 2–DE allows the separation of 3,000 proteins in a mixture, which can be visualized as discrete spots by staining with a dye (see figure 2) (Link 1999). Two-dimensional gel electrophoresis was used to derive several databases of human proteins found in body fluids or in different cell types that are associated with certain diseases (Merril et al. 1995; Lemkin et al. 1995; Celis et al. 1996). However, 2–DE also has its limitations. For example, this method cannot reveal low-abundance proteins because a minimum amount of protein has to be present to be detectable, and low-abundance proteins may be lost during sample fractionation (Gygi et al. 2000). Further, a single spot on a 2–DE gel can contain one abundant protein and several low-abundance proteins that have not separated from each other because they are similar in size and charge.

#### MS

**Figure 3:**
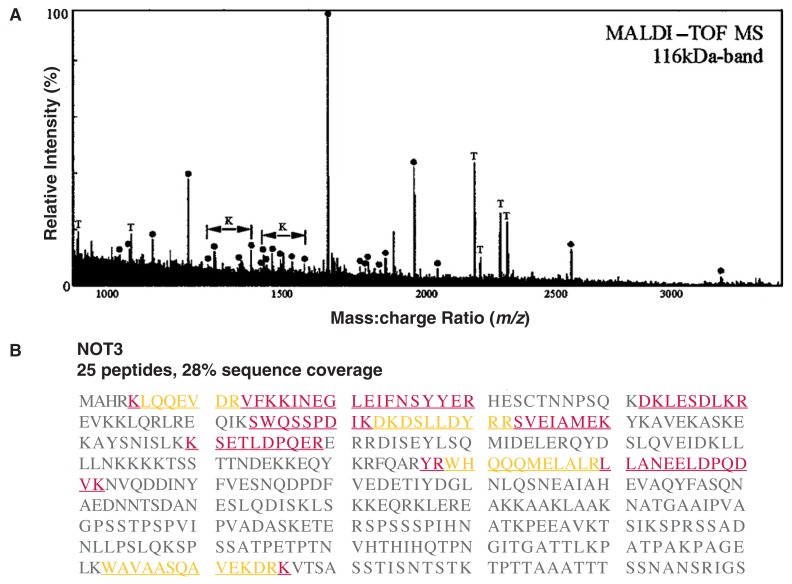
(A) Example of a matrix-assisted laser desorption/ionization time-of-flight (MALDI–TOF) peptide mass spectrum. Identified trypsin-derived peptides are marked with a filled circle. This analysis identified a total of 25 tryptic peptides with a mass:charge ratio (*m/z*) between 800 and 4,000 Da. (B) A graphical representation of the result of a database search showing the protein identified. Peptides that matched the sequence of that protein appear in dark red, and sequences that were covered by overlapping peptides are shown in yellow. The 25 identified peptides cover 28 percent of the protein sequence. The data obtained suggest that the protein being studied is the protein identified in the database search. SOURCE: Reprinted from Mann et al. 2001, with permission of the authors.

In proteomics studies, 2–DE and HPLC are combined with MS in order to identify the protein(s) present in each gel spot or buffer fraction, respectively. (See figure 1 for a summary of this process.) For example, when a sample (e.g., a protein extract derived from a certain tissue, cell type, organelle, or other cellular component) has been separated by 2–DE, a spot of interest is cut out of the gel. The protein(s) in that gel spot are degraded with the help of a protease—an enzyme that cleaves proteins into peptides at specific sites—such as the commonly used trypsin. This process is called proteolysis. Most proteins yield at least 20 fragments (i.e., tryptic peptides) after being digested with trypsin. Next, the molecular weights of the tryptic peptides are determined with high accuracy using a technique called matrix-assisted laser desorption/ionization time-of-flight mass spectrometry (MALDI–TOF MS). In brief, in this technique, the peptide mixture is combined with a matrix material and ionized by a laser beam. The ionized peptide molecules then travel according to their mass through a tube to a detector. For two ions with equal charges, the lighter one will reach the detector faster than the heavier one. The detector calculates a mass:charge ratio of each ionized peptide; each peptide is displayed as a peak on a printout or screen (see figure 3A). The computer then generates a list of all measured peptide masses in a peptide mixture. This list is compared with the masses of peptides that would be expected after theoretically digesting all known proteins in databases with trypsin to see if the protein analyzed has already been identified (see figure 3B). For a positive identification, the masses of at least five peptides from the unknown protein should match those of a known protein, and these peptides combined must cover at least 15 percent of the protein sequence.

Protein identification based on peptide masses obtained with MALDI–TOF MS is called peptide mapping, or peptide mass fingerprinting analysis. It is the primary analytic approach because it can quickly and accurately analyze small amounts of complex protein mixtures. For organisms whose genome is fully known (and for which one can therefore deduce the sequence of most proteins), researchers typically can unambiguously identify 50 to 90 percent of the proteins detected by 2–DE using MALDI–TOF MS peptide mapping. Overall, however, 2–DE combined with MALDI–TOF MS can detect only 20 to 30 percent of all the proteins in a mixture. One reason for this low efficiency is that 2–DE usu ally cannot separate proteins with low abundance, proteins found in mem branes, and proteins of similar molec ular weight or isoelectric point. Moreover, some tryptic peptides do not ionize well and therefore cannot be detected by MALDI–TOF. In these cases, a technique called elec tronspray ionization (ESI) combined with two or more steps of MS (MS^n^) can be used to determine first the peptide masses and then the amino acid sequence of the most abundant peptides (see figure 1). In ESI, parent tryptic peptides are gently ionized in solution; fragmented into smaller pieces, so-called daughter ions; and transferred to an ion-trapping mass spectrometer. The first mass-analyzing step selectively separates the parent ions, and the second step analyzes the fragmented daughter ions of a selected parent ion. This method generates information about peptide masses that can be compared with the information on known proteins and their corresponding peptides available in sequence databases, as well as fragmentation patterns that provide sequence information.

Although 2–DE data have produced several databases of human proteins in body fluids and different cell types associated with diseases (Merril et al. 1995; Lemkin et al. 1995; Celis et al. 1996), the proteins affected in pathological conditions are usually not the most abundant ones. Using a new HPLC-ion trap MS^3^ system, investigators were able to detect a low-abundance protein—human growth hormone—in tryptic digests of plasma proteins (Wu et al. 2001). The difficulty of such analyses arises from the fact that the concentration of human growth hormone (16 femtomoles) is only one forty-thousandth of the total plasma protein. These concentration differences can be even greater for other proteins. Accordingly, numerous technological developments and enhancements have emerged so that MS can characterize low-abundance proteins, even without enrichment by chromatographic methods.

As an alternative for the fast identification of a great number of proteins, researchers at Indiana University (Valentine et al. 1998) have developed a new system called ion mobility MS (IMMS), which in one step performs electrophoresis and MS of peptides. In combination with multidimensional chromatography, IMMS identified 70 to 90 percent of the proteins in a sample, compared with only 20 to 30 percent detected by traditional 2–DE and MALDI–TOF MS, as described above. This novel system holds great promise for fast identification especially of unknown proteins.

### Mass Spectrometry for PTMs

Repeated MS steps are necessary to characterize proteins with PTMs because these chemical groups are often difficult to remove to reveal their attachment sites on the proteins. Despite the technical difficulties caused by the assortment of PTMs, investigating these modifications is important because they contribute to the eventual structure and function of many proteins and might affect how the modified proteins interact with other cellular molecules. Moreover, investigators can gain critical information by determining the presence and role of different PTMs in a variety of developmental and pathophysiological conditions.

The phosphoproteome, which consists of all phosphorylated proteins, has attracted particular attention because phosphorylated proteins play important roles in signal transduction pathways that communicate events occurring at the cell’s surface into the cell and its compartments. To analyze these proteins, researchers can use two main approaches: They can either separate phosphorylated peptides from a peptide mixture using a procedure called affinity chromatography (Oda et al. 2001), or they can compare phosphorylated with nonphosphorylated samples after the removal of the phosphate groups.

Another approach to detecting PTMs is a technique called ESI/Fourier transform MS, which can directly fragment medium-sized proteins instead of just smaller tryptic peptides. In an ambitious study, Meng and colleagues (2001) used this approach to directly identify intact proteins rather than their tryptic peptides in a complex cellular mixture after two-dimensional LC. Small proteomes, such as that of a bacterium with about 700 proteins, can be successfully mapped in this fashion, and the methodology could be extended in the future and applied to larger proteomes.

### Mass Spectrometry for Protein Quantification

Besides identifying proteins in a mixture and their PTMs, researchers must analyze how much of a given protein is present in order to understand the effects of drugs, disease, developmental factors, and external stimuli on protein levels. These quantitative analyses can be accomplished by comparing proteins from two different states (e.g., before and after a tissue has been exposed to alcohol) in the same setting. The proteins from each state are labeled by adding different fluorescent tags or radioactive molecules called isotope-coded affinity tags (ICAT). The two samples are then mixed and separated on a single gel by 2–DE—a process known as difference gel electrophoresis (DIGE) (Naaby-Hansen et al. 2001; Peng and Gygi 2001). With repeated MS of ICAT–DIGE-separated proteins, one can determine the relative expression of component proteins in large, complex samples, including low-abundance proteins not detected with conventional 2–DE. Using a combination of ICAT, multidimensional LC, and MS, Han and colleagues (2001*a*) determined the ratios of 491 microsomal proteins in 2 cellular states. In the field of alcoholism, the ICAT/MS tools will play a pivotal role in studying differences in protein expression and for discovering diagnostic markers as discussed in the section “What Is Ahead for Alcohol Research?” below.

### Protein–Protein Interactions

Interactions among proteins are necessary for almost every physiological process, from maintaining the shape of the cell with a mesh of structural proteins to sensing extracellular signals and transmitting them into the cell. Protein coordination also is required for performing specialized jobs, such as breaking down drugs and providing oxygen to tissues. By conducting “fishing expeditions” using a known component as “bait,” researchers try to link proteins into common biological functions and cellular processes. This section describes some of the methods used in these analyses.

#### Two-Hybrid Screens

**Figure 4:**
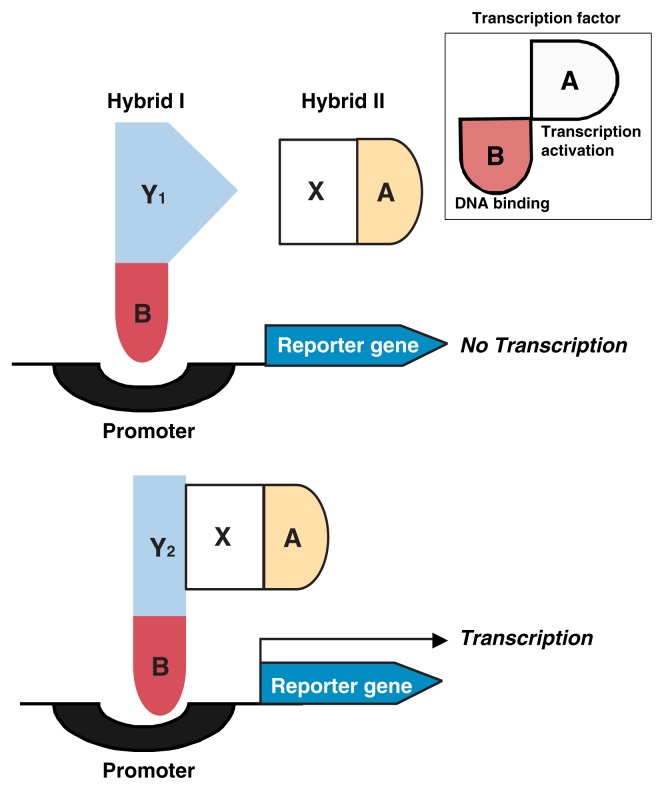
Schematic representation of the principle underlying the two-hybrid system for detecting in vivo protein–protein interactions. The assay is based on the fact that the transcription of the reporter gene is regulated by the activity of a specific protein (i.e., a transcription factor). Transcription factors are modular proteins consisting of two domains, a DNA-binding domain B and an activating domain A (see inset). To test if a known protein X interacts with a series of proteins Y (e.g., Y_1_, Y_2_, etc.), fusion proteins are genetically engineered in which domain A is fused to X (hybrid II) and domain B is fused to the Y proteins (hybrid I). Neither domain in the hybrid molecules thus generated can activate transcription alone if proteins X and Y do not interact (see upper panel). Only if proteins X and Y interact can domains A and B come close together so that the reporter gene can be transcribed (see lower panel). A similar approach can also be used to screen for complex interactions of three proteins (i.e., three-hybrid system) or for interactions between a protein and nucleic acids (i.e., one-hybrid system).

The most widely used, albeit laborious method of determining whether two proteins interact is the two-hybrid genetic system (figure 4). It is based on the fact that, in higher organisms, proteins regulating gene expression (i.e., transcription factors) consist of two modular parts. One part, called the binding domain (B), interacts with a DNA segment (i.e., the promoter area) near the gene whose activity is being regulated. The other part, called the activating domain (A), interacts with an enzyme that helps generate mRNA molecules from DNA. This mRNA subsequently serves as template for translation and protein production. Through genetic engineering one can separately produce the two domains of a transcription factor and fuse each part to a different protein, thus creating hybrid proteins. For example, one can couple domain A to a known protein X and domain B to a series of other potential binding proteins (Ys). Neither by themselves nor fused with their respective attached proteins can the A domain and B domain activate a test gene in a host yeast or mammalian cell. Only when the A and B domains are brought together—because the proteins X and Y coupled to them interact with each other—is the hybrid complex A–X:Y–B formed, which can activate gene transcription. One can then identify host cells that contain the functional hybrid complex, isolate the specific Y protein that was fused to domain B, and study it further. Using a large-scale yeast 2-hybrid system, Uetz and colleagues (2000) demonstrated 957 putative protein–protein interactions involving 1,004 of the 6,000 yeast proteins tested.

#### From Protein Pairs to Networks

Once pairs of interacting proteins are discovered, the next step is to link them into complexes, pathways, and networks. The exquisite complexity of protein–protein interactions is now emerging. The networks of interacting proteins can be compared to large maps of airline routes. A single protein can interact with several other proteins, much as one airline can fly several routes from a single hub. Of these interactions, “local” protein connections are relatively easy to understand because the interacting proteins are often located in the same compartment of a cell or share a common metabolic pathway. For example, proteins that control the cell cycle interact predictably with proteins involved in cell division, DNA synthesis (required for cell division), and amino acid metabolism (needed for new protein synthesis). Other interactions represent “long-distance” connections—for example, pathways that link proteins regulating the cell cycle and proteins involved in signal transduction. Detailed analyses of protein interactions can uncover highly complex networks of interacting proteins. Tucker and colleagues (2001) have assembled an extended network map of about 1,200 protein–protein interactions in yeast.

Detailed analyses of protein interactions can also uncover the roles of so-called orphan proteins—proteins that previously had no assigned function. Similarly, researchers will likely identify sets of abnormal interactions or the absence of established interactions that are associated with the development of diseases. Finally, investigators can screen computer databases (i.e., conduct *in silico* analyses) of established networks from model organisms (Walhout at al. 2000; Rain et al. 2001) to identify potential partners and therefore provide clues about the functions of proteins under investigation in other organisms.

Data from such interaction studies must be evaluated carefully, however, to avoid misleading conclusions. For example, investigators must determine the localization of unknown proteins in the cell in order to exclude apparent interactions that have no biological significance because the proteins involved are located in completely different cell compartments (i.e., false positives). Similarly, one must be aware of the possibility of false negatives—failures to detect interactions because other cellular molecules impede protein–protein interactions in the experimental system. False negatives also may result when proteins are not expressed properly (e.g., when they assume an abnormal three-dimensional structure or fail to localize to the correct cell compartment under experimental conditions). Protein arrays, described in the next section, avoid these pitfalls by directly analyzing protein–protein interactions.

#### Protein Arrays

Protein arrays or microarrays, also known as protein chips, are the latest addition to the proteomics toolkit. These chips are stamp-sized surfaces that are coated in a dense and ordered manner with minute amounts of several thousand proteins. Each protein on an array can be identified by its spatial coordinates. Nowadays, commercially available microarrays carry up to 1,000 proteins. Human proteome chips carrying as many as 100,000 proteins may be available in coming years.

The simplest arrays carry antibodies—proteins generated by the immune system of vertebrates in response to the presence of foreign molecules in the body. Each antibody can recognize and interact with one or more specific molecules, thereby marking those molecules as “foreign” and targeting them for destruction. In the laboratory, antibodies are commonly used as probes to profile and quantify patterns of protein expression in a sample. By incubating an array that contains antibodies to known proteins with a protein extract, one can determine which of the proteins are present in the extract.

To identify and quantify protein– protein interactions in arrays, researchers attach certain molecules (i.e., “tags”) to the proteins on the array. These tags fluoresce only after protein–protein interactions have been established. Ideally, each protein should get a tag with its individual color, like a product barcode, so that one can immediately determine which proteins participate in multiple interactions. To achieve this goal for complex proteomes with huge numbers of proteins, one can employ a new technology using fluorescent semiconductor nanoparticles called quantum dots (Alivisatos 2001; Han et al. 2001*b*). These dots can provide a rainbow of theoretically billions of distinctive bright colors to code all known proteins. Quantum dots can thus allow for simultaneous measurement of many samples, even in solution rather than on a solid microarray.

**Table:** List of Web Sites Related to Proteomic Analyses

Web site	Content
http://www.hupo.org	This is the site of the Human Proteome Organization with links to databases and tools.
http://www.ncbi.nlm.nih.gov	The National Center for Biotechnology Information hosts several databases and tools for data mining.
http://www.proteome.com	The site, which is sponsored by Incyte, provides information on the structure, functions, and interactions of proteins of yeast, worm, mouse, rat, and human.
http://www.expasy.ch/	The Expert Protein Analysis System proteomic server offers access to databases and several proteomic software tools.
http://image.llnl.gov/	This site was developed by the Integrated Molecular Analysis of Genomes and Expression Consortium, and is a public source of cDNA clones.
http://www.spectroscopynow.com	This useful resource offers educational material and the latest developments in proteomic mass spectroscopy.
http://bioinformatics.org	This site provides bioinformatics resources, such as a glossary, tutorials, practical tips, and computational tools.

One can also conduct antibody-based assays in the reverse format, with proteins located on an array. These assays are much faster to perform than 2–DE or LC, and they are more powerful than other assays based on reactions between proteins and antibodies (i.e., single immuno-assays). Because antibodies are pivotal tools in the study of arrayed proteins as well as in other applications and because the number of antibodies available is still limited, the Human Proteome Organization (HUPO) made the availability of an antibody for every human protein its top priority. HUPO is the counterpart to the Human Genome Organization and is devoted to deciphering the human proteome. (For the Web site of HUPO and other Web sites related to proteomic analyses, see the table.) By using genetic engineering rather than the traditional laborious use of animals, antibody production could be simplified by novel technologies such as phage display, a technique that uses bacterial viruses to generate foreign peptides or proteins, includ ing highly specific antibodies similar to those found in humans (Li 2000).

**Figure 5:**
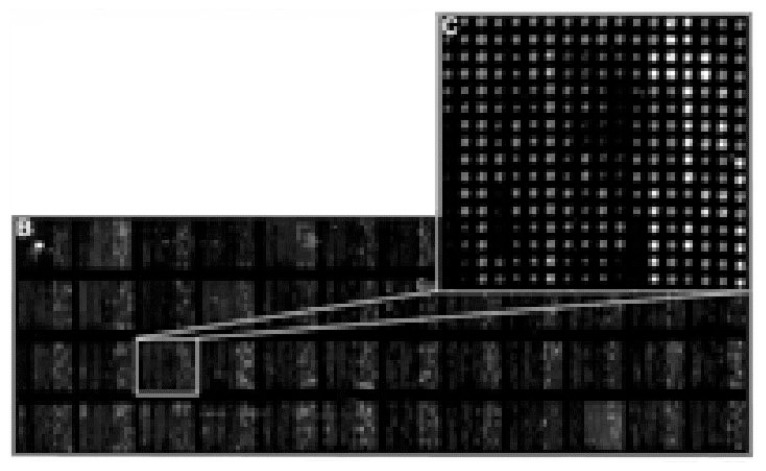
Example of a proteome microarray carrying 5,800 unique yeast proteins, which represents the entire yeast proteome. The enlarged area shows one of the 48 blocks containing 288 protein dots each. A minimum of 10 femtograms of protein is deposited per dot and detected as bright color (the lighter dots). The yeast proteome in the microarray is further tested for protein–protein interactions with known proteins of interest that carry another fluorescent color. NOTE: One femtogram is 10^−15^ g. SOURCE: Redrawn from Zhu et al. 2001, with permission of the authors.

Other protein arrays carry peptides that are generated using the phage-display technique. Using phages, one can generate combinatorial peptide libraries—large collections of diverse peptides that are produced by systemat ically assembling protein building blocks in as many combinations as possible. Peptides are then screened, and specific ones are chosen that are part of the interacting sites of proteins and that recognize unique sites on proteins (e.g., domains that typically interact with other proteins). Such peptide arrays can be used as surrogates for antibodies to “fish out” partner proteins from com plex protein mixtures. Finally, protein arrays can carry collections of purified proteins that are to be analyzed further. For example, researchers recently spot ted the complete set of yeast proteins on a chip and analyzed interactions with several key proteins (see figure 5) (Zhu et al. 2001).

One of the major technical diffi culties associated with protein chips is the application of a mixture of pro teins. When proteins randomly attach to the chemically modified array surfaces, their three-dimensional structures may become distorted, resulting in inactivation or instability of the proteins. To avoid this prob lem, one can add short peptides or other small molecules to the arrays that serve to anchor the proteins in an undisturbed, oriented fashion. When this is not feasible, one can also immobilize proteins on a thin glass slide coated with a thin layer of a gel-like material that provides a solution-like environment and therefore does not distort protein structure.

Other challenges associated with protein chips are how to keep the sample volume to a minimum, gener ate a high density of proteins on the array, ensure uniformity of various arrays, enhance the range of signal linearity, increase signal intensity of specific protein–protein interactions, and minimize signals from nonspe cific interactions (i.e., background signals). Additional efforts are aimed at designing techniques to detect binding reactions between biological structures larger than proteins (e.g., between cancerous cells or between cells and viruses or other disease-causing organisms).

### Data Analysis

Processing of proteomic samples is cur rently performed at best in a parallel fashion. However, in order to achieve high-throughput—that is, massive par allel screening for the simultaneous anal ysis and evaluation of a large number of samples—several steps in the sample analysis and evaluation of results should be standardized through automation. This automation can be achieved by establishing online procedures in which various instruments are physically connected and computer-controlled. One instrument then directly feeds the processed sample to the subsequent instrument, and operator intervention is not needed. The instrumentation of HPLC and ESI– MS is amenable to such online operation. In contrast, offline procedures require an operator to handle samples and manually feed instruments, slowing down sample processing and creating bottlenecks.

Intelligent data-dependent acquisition by computers is also necessary for reducing the size of data collection. For example, additional MS steps should be performed only for peptides of relative high abundance until proteins are unambiguously identified.

Given the enormous amounts of raw data on ion masses and fragments generated by MS, automated database searches (i.e., data mining) and data interpretation must be employed to reassemble, like a jigsaw puzzle, the sequences of the peptides, the proteins from which the peptides were derived, and their PTMs. Such calculations have become realistic with the availability of supercomputers and the boom in the bioinformatics field (Misener and Krawetz 1999).

Data mining—the (semi−)automated search for relationships and global patterns within data—also is essential for proteomics analyses, including protein array analyses. For example, one must normalize array data to allow for comparisons either between two samples or across repeated experiments. In addition, researchers must be able to distinguish real biological changes from nonspecific experimental variations and to find patterns and groupings in the observed variations that correlate with biological function (e.g., proteins categorized by response to acute or chronic alcohol exposure). Some of the currently available bioinformatics tools of data mining have been developed for handling genomic data and must be reinvented for proteomics analyses.

## What Is Ahead for Alcohol Research?

### Genomic Leads

Although no direct applications of proteomic research to the alcohol field have been reported, some leads may come from earlier genomic projects. In a recent study, Xu and colleagues (2001) compared gene expression in the brains of mice that are greatly sedated by alcohol (i.e., long-sleep mice) and mice that are resistant to alcohol’s sedative effects (short-sleep mice). Using DNA microarrays carrying up to 18,000 genes, the investigators identified 41 genes whose expression in the brain differed significantly between the 2 strains. Future studies may help characterize the functions and interactions of the proteins encoded by those genes as well as their localization in particular brain areas. Direct proteomic studies of different brain cells of animal models will be instrumental for understanding the mechanism of alcohol’s sedative effects.

In another genomic study, Thibault and colleagues (2000), using arrays representing 6,000 genes of cultured human nerve cells, detected a set of 42 alcohol-responsive genes. Most pronounced was an increase in the expression of three genes involved in the production of a brain chemical (i.e., neurotransmitter) called norepinephrine. This increase correlated with the amounts of the respective proteins. However, the products of six other genes that were modulated by alcohol remain unknown. The knowledge gained from this study may be channeled into proteomics research. For example, by directly analyzing the proteome of human nerve cells one may obtain information about the proteins encoded by these unknown genes. Moreover, *in silico* analyses of protein networks of model organisms with the unknown human gene products may provide clues about the structure, function, and interactions of the human gene product. Through such approaches, proteomic analyses could elucidate the mechanisms underlying alcohol’s toxic effects in the brain and the development of alcohol dependence and addiction.

Another genomic study investigating expression of 4,000 genes in postmortem brain samples from a brain region called the superior frontal cortex area of alcoholics and nonalcoholic control subjects (Lewohl et al. 2000) found that 163 genes differed by about 40 percent between alcoholics and nonalcoholics. Of particular interest is the fact that the expression of genes related to the production of myelin—a molecule that is wrapped around certain parts of nerve cells (i.e., the nerve axon) as an insulation and which gives the white matter of the brain its characteristic color—was reduced in alcoholics. A loss of cerebral white matter has previously been observed in alcoholics and in children with fetal alcohol syndrome, a finding that may extend alcohol’s effects on myelin-related proteins to other brain regions. Overall, however, comparatively few of the genes tested were affected by long-term alcohol abuse in humans (163 out of 4,000, or 4 percent), and a difference in gene expression of 40 percent between alcoholics and nonalcoholics is rather small.^1^ These findings are similar to those reported in studies of aging and suggest that the brain may adapt to chronic alcohol exposure. By expressing these proteins in genetically engineered cells and studying them on protein chips, researchers could unravel some of the mechanisms underlying alcohol’s neurotoxic effects on the brain.

Finally, cutting-edge genomic research aims to analyze changes in global gene expression in response to alcohol exposure, using single-type cells excised by a technique called robotic laser capture microdissection from various tissues (e.g., brain or liver). These genomic studies are expected to point out important groups of proteins that should be analyzed using protein chips in order to uncover how different types of cells in different organs adapt to the presence of alcohol. Technologies currently being developed to measure changes in protein levels directly by MS methodology also could be used in the alcohol field.

### Alcoholomics

The term “alcoholomics” refers to the study of those proteins (i.e., the subproteome) that are directly or indirectly affected by alcohol. Four areas in alcohol research would greatly benefit from proteomic studies: (1) identification of biomarkers of alcohol-related characteristics (i.e., phenotypes) and alcohol-related diseases, (2) quantification of biomarker levels, (3) PTMs associated with alcohol-related biomarkers, and (4) discovery of novel drug targets and innovative medications.

#### Biomarkers

Biomarkers are defined as specific molecules or molecular changes that are associated with biological functions and whose presence or absence is indicative of those functions. Two types of biomarkers are sought in the alcohol field: diagnostic and prognostic. Diagnostic biomarkers detect diseased tissue at the earliest stage of disease progression, when other detection methods fail; prognostic biomarkers can indicate the disease stage and foretell disease outcome. Proteomic applications have had great success in identifying prognostic and diagnostic biomarkers for several diseases, including cancer (e.g., liver and prostate cancer), cerebral palsy, severe combined immunodeficiency, and autism.

To identify alcohol-related biomarkers, researchers must compare protein expression in biological fluids and tissues of alcoholic and nonalcoholic human subjects or experimental systems (i.e., animal models or cultured cells). For example, one can look for prognostic markers of excessive alcohol consumption by comparing protein expression in animal models that have been bred to display specific alcohol-related behaviors (e.g., mice or rats that are sensitive or insensitive to alcohol’s sedative effects or that exhibit different levels of preference for alcohol). Those proteins that by MS or protein array technology are shown to differ between the alcoholic and nonalcoholic samples can potentially become useful clinical biomarkers. For example, Kristensen and colleagues (2000) analyzed the proteome of rat hepatic stellate cells. These are specialized fat-storing liver cells that normally are inactive (i.e., quiescent) and whose activation is a key event in early stages of liver injury (i.e., fibrosis). Using a combination of 2–DE plus ESI–MS^2^, the investigators identified a total of 156 stellate proteins, 43 of which were affected by cell activation. Even such partial knowledge of differences in the stellate proteome between quiescent and activated states could contribute to diagnostic tools, such as a chip carrying antibodies recognizing the key proteins found to be uniquely present (or absent) at the onset of fibrosis in humans.

#### Quantification of Protein Levels

To diagnose or determine the stage of a disease, it is often essential to determine not only the presence or absence but also the specific levels of certain proteins. In alcoholism research, the ICAT/MS approach will play a pivotal role in allowing researchers to detect quantitative differences in protein expression—for example, by comparing protein levels between tissues (e.g., liver, heart, and brain), cell types (e.g., various white blood or brain cells), or biological fluids (e.g., serum,^2^ bile, and urine) of control and alcoholic samples. Antibody chips, as described in the previous paragraph, that identify alcohol-related biomarkers could also quantify these proteins. However, these analyses also have to consider that in many cases several factors (e.g., alcohol, toxins, viruses, and cancer) can cause the same effect (e.g., increases in levels of liver proteins). To generate more specific results, it therefore would be preferable to evaluate a panel of alcohol-related biomarkers and their levels rather than just one protein.

#### PTMs

Investigation of PTM-based biomarkers of alcoholism also may yield exciting results. As mentioned earlier, one of the most common PTMs is protein phosphorylation, which modulates the activity of signal transduction pathways. Proteomic analyses may help elucidate how alcohol perturbs such pathways. For example, using a combination of one-dimensional electrophoresis, LC, and MS, Pandey and colleagues (2000) identified the key players in a signal transduction pathway initiated by a molecule called epidermal growth factor. In alcohol research, studies could focus on analyzing differences in the phosphorylation of critical proteins of such cellular pathways between alcoholic and control samples. Such analyses could be accomplished with the help of antibody chips that will help determine the degree of phosphorylation for each protein of interest, the ratio of phosphorylation versus nonphosphorylation for individual proteins, and additional information on the site where phosphorylation occurs on the protein.

Another PTM relevant to the alcohol field is the addition of sialic acid to transferrin, a protein secreted from the liver into the blood. Studies found that sialic acid levels are significantly lower in alcoholics than in nonalcoholic patients (Sanchez et al. 1995). The observation that chronic alcohol consumption inhibits the incorporation of sialic acid into transferrin and other glycoproteins has been used in a laboratory test for chronic alcohol abuse (Anton 2001).

Direct products of alcohol metabolism (e.g., alpha-hydroxyethyl radicals, acetaldehyde, and lipid peroxides) also cause PTMs that correlate with alcohol consumption in studies of animal models and human subjects. In earlier efforts, researchers identified the protein sites where these PTMs occur. Most recently, using MALDI– TOF MS in combination with HPLC, investigators determined the protein sites where the alpha-hydroxyethyl radical was preferentially attached (Anni and Israel 1999) and identified peptides produced by phages that recognize this PTM on proteins (Anni et al. 2001*a*). In addition, researchers are trying to determine which proteins are particularly prone to alcohol-induced PTMs. These efforts have already led to the identification of some proteins, and new ones are being discovered. For example, acetaldehyde was found to modify a molecule called cysteinyl-glycine,^3^ resulting in the formation of a compound called 2-methyl-thiazolidine-4-carbonylglycine, which was identified by HPLC combined with ESI-MS^4^ (Anni et al. 2001*b*). This new molecule was found in the bile of rats after alcohol intoxication, and its presence in other biological fluids might be indicative of chronic alcohol consumption.

Proteomic analyses using antibodies specific for PTMs caused by alcohol metabolites in combination with antibodies against proteins found in the serum may help researchers and clinicians identify those proteins that have been modified by alcohol, as well as the ratios of modified versus unmodified proteins by screening control and alcoholic samples. If one could correlate the presence of such PTMs in serum proteins with liver proteins, it would be possible to develop a noninvasive diagnostic tool for detecting alcohol-related liver damage based on the direct effects of alcohol products. Such a test would be highly specific for the early identification of people with drinking problems, and it could easily be combined on a chip with tests for other alcohol-relevant PTMs.

### Drug Targets and Drug Discovery

Proteomic studies also could lead to the identification of proteins that can serve as novel targets for medications or to the development of new medications. For example, studies like that of the proteome of stellate cells mentioned earlier could yield protein targets for effective medications to treat the fibrosis of the liver caused by alcohol or other factors. Similarly, researchers may want to study the proteome of a type of immune cell called macrophages, which are found in the blood and in the liver (where they are referred to as Kupffer cells). These analyses might identify critical proteins involved in early signaling pathways induced by alcohol, which may lead to liver damage. Currently, fewer than 500 proteins have been identified as potential targets for drug development, and studies of the proteomes of specific cell types would certainly increase this number substantially.

Novel medications for alcohol-related problems also could be derived by proteomic and the previously mentioned phage-display technologies. Using phages, one can generate combinatorial peptide libraries which are a rich source of molecules that can activate or inhibit receptors or enzymes, inhibit protein assembly or protein–protein interactions, or serve as antibodies. One could then use the peptides in a library as probes on a chip carrying proteins that could serve as potential medication targets. Those peptides from the library that interact with the potential target proteins could be identified and studied further for possible development into new medications (Anni et al. 2002). Peptides or antibodies generated by phages and identified through proteomic approaches also could act to bind or block the binding of a drug to its target proteins (e.g., after a drug overdose) or to improve the transport of a medication to its site of action to increase its potency/specificity and decrease side effects (e.g., “magic bullets” for liver cancer). The possibilities for proteomics-related breakthroughs in the alcohol field are comparable with the potential benefits of this approach in other fields.

## Conclusions

The field of proteomics has yet to be applied consistently in alcoholism research, but its enormous potential most likely will be tapped soon. Numerous proteomic technologies (e.g., multidimensional electrophoresis and LC, tandem MS, hybrid screens, protein arrays, and phage display) are already available. Although some leads from genomic research could form the basis for proteomic studies in the alcohol field, non-hypothesis-driven proteomics research is poised to identify a novel set of molecules on which investigators can focus as potential diagnostic or therapeutic targets. Through such analyses, proteomics will eventually become indispensable and complement genomic analyses of alcoholism. Proteomics will help delineate the mechanisms underlying alcohol-related tissue injury, morbidity, dependence, and withdrawal symptoms as well as advance diagnostic and prognostic tools. Moreover, proteins are a potential gold mine for the discovery of new drug targets and therapeutic interventions. The greatest difficulties in utilizing these opportunities will be to determine research priorities and to develop appropriate model systems and bioinformatics tools for proteomic data mining.

## Footnotes

1 For comparison, the differences in gene expression between healthy and cancerous cells are five- to tenfold instead of 40 percent.

2 The serum is the clear, fluid component of the blood that remains after all blood cells and certain other molecules have been removed.

3 Cysteinyl-glycine is naturally present in the bile and is derived from glutathione, which is an important antioxidant liver tripeptide.
